# Microdosimetric Analysis Confirms Similar Biological Effectiveness of External Exposure to Gamma-Rays and Internal Exposure to ^137^Cs, ^134^Cs, and ^131^I

**DOI:** 10.1371/journal.pone.0099831

**Published:** 2014-06-11

**Authors:** Tatsuhiko Sato, Kentaro Manabe, Nobuyuki Hamada

**Affiliations:** 1 Research Group for Radiation Protection, Japan Atomic Energy Agency (JAEA), Shirakata Shirane 2-4, Tokai, Ibaraki, Japan; 2 Radiation Safety Research Center, Nuclear Technology Research Laboratory, Central Research Institute of Electric Power Industry (CRIEPI), 2-11-1 Iwado-kita, Komae, Tokyo, Japan; University of California Davis, United States of America

## Abstract

The risk of internal exposure to ^137^Cs, ^134^Cs, and ^131^I is of great public concern after the accident at the Fukushima-Daiichi nuclear power plant. The relative biological effectiveness (RBE, defined herein as effectiveness of internal exposure relative to the external exposure to γ-rays) is occasionally believed to be much greater than unity due to insufficient discussions on the difference of their microdosimetric profiles. We therefore performed a Monte Carlo particle transport simulation in ideally aligned cell systems to calculate the probability densities of absorbed doses in subcellular and intranuclear scales for internal exposures to electrons emitted from ^137^Cs, ^134^Cs, and ^131^I, as well as the external exposure to 662 keV photons. The RBE due to the inhomogeneous radioactive isotope (RI) distribution in subcellular structures and the high ionization density around the particle trajectories was then derived from the calculated microdosimetric probability density. The RBE for the bystander effect was also estimated from the probability density, considering its non-linear dose response. The RBE due to the high ionization density and that for the bystander effect were very close to 1, because the microdosimetric probability densities were nearly identical between the internal exposures and the external exposure from the 662 keV photons. On the other hand, the RBE due to the RI inhomogeneity largely depended on the intranuclear RI concentration and cell size, but their maximum possible RBE was only 1.04 even under conservative assumptions. Thus, it can be concluded from the microdosimetric viewpoint that the risk from internal exposures to ^137^Cs, ^134^Cs, and ^131^I should be nearly equivalent to that of external exposure to γ-rays at the same absorbed dose level, as suggested in the current recommendations of the International Commission on Radiological Protection.

## Introduction

The risk of internal radiation exposure is of great public concern after the accident at the Fukushima-Daiichi nuclear power plant [1], [2]. This is partially because the risks from internal exposure can differ from those from external exposure to γ-rays at the same absorbed dose level, i.e., their relative biological effectiveness (RBE, defined herein as effectiveness of internal exposure relative to the external exposure to γ-rays) is not always 1. For example, there is evidence that RBEs for the intake of α, low-energy β, and Auger-electron emitters are greater than 1 [3], [4]. In general, the high ionization density around the trajectories of α particles and low-energy electrons as well as the inhomogeneous radioactive isotope (RI) distribution in subcellular structures are considered to explain the higher RBE values. These are referred to hereafter as the track-structure and RI-inhomogeneity effects, respectively.

A number of studies have been carried out to estimate the RBE for the intake of α, low-energy β, and Auger-electron emitters [5]–[7], as these results are well summarized in Report of the Committee Examining Radiation Risks of Internal Emitters [4]. In contrast, the RBE for the intake of ^137^Cs, ^134^Cs, and ^131^I (major contributors to the internal exposure dose from the nuclear accident in Fukushima) was not extensively discussed. This is because these RIs emit relatively high energy electrons and photons, and because the scientific community considers that their RBE is 1. Nevertheless, the public occasionally believes that the risk from internal exposure is much greater than that from external exposure even for the intake of ^137^Cs, ^134^Cs, and ^131^I, albeit no supportive scientific evidence. Such belief comes, at least in part, from the lack of a detailed analysis of the contribution of the track-structure and RI-inhomogeneity effects to the RBE for the intake of these RIs, except for the RI-inhomogeneity effect of ^131^I [8]. The contribution of these effects may not be negligible, if these RIs are selectively located inside cell nucleus. An animal study has suggested that 12–21% of ^137^Cs are localized in cell nuclei [9].

We therefore set out to quantitatively analyze the contribution of the track-structure and RI-inhomogeneity effects for the intake of ^137^Cs, ^134^Cs, and ^131^I, and for this, a microdosimetric simulation was performed using the Particle and Heavy Ion Transport code System (PHITS) version 2.64 [10]. The RBE for internal exposure to these RIs was then derived as a function of cell size and the fraction of the RI distributed in cell nuclei. In addition, the RBE for the bystander effect (biological effect caused by signaling from irradiated to non-irradiated cells) [11], [12] was also discussed based on the inhomogeneity of absorbed dose among cell nuclei, since the bystander effect may play an important role in the risk estimation of low-dose internal exposure owing to its non-linear dose response [13], [14]. The results of the simulation, together with the maximum possible RBE from the dosimetric viewpoint, are presented in this paper.

## Materials and Methods

### Monte Carlo Particle-transport Simulation in Cell Systems

We performed Monte Carlo particle transport simulations in ideally aligned cell systems that were internally exposed to electrons emitted from ^137^Cs, ^134^Cs, and ^131^I, using the PHITS code. Electrons emitted from ^137m^Ba, which is a daughter isotope of ^137^Cs with a half-life of 2.552 min, were also considered in the simulation of the intake of ^137^Cs. Similar simulations were also performed for internal exposures to electrons from ^3^H, α particles from ^239^Pu, and 662 keV mono-energetic photons, which is the dominant γ-rays from ^137^Cs (in strict sense, 662 keV photons are emitted from the decay of ^137m^Ba). The last simulation condition also represented the external exposure to γ-rays, because the source location is not an important factor for photon exposure from the microdosimetric viewpoint. Thus, this condition served as the reference condition in this study, i.e., its RBE is equal to 1. The energy spectra of particles emitted from the RIs were taken from the International Commission of Radiological Protection (ICRP) Publication 107 [15].

**Figure 1:**
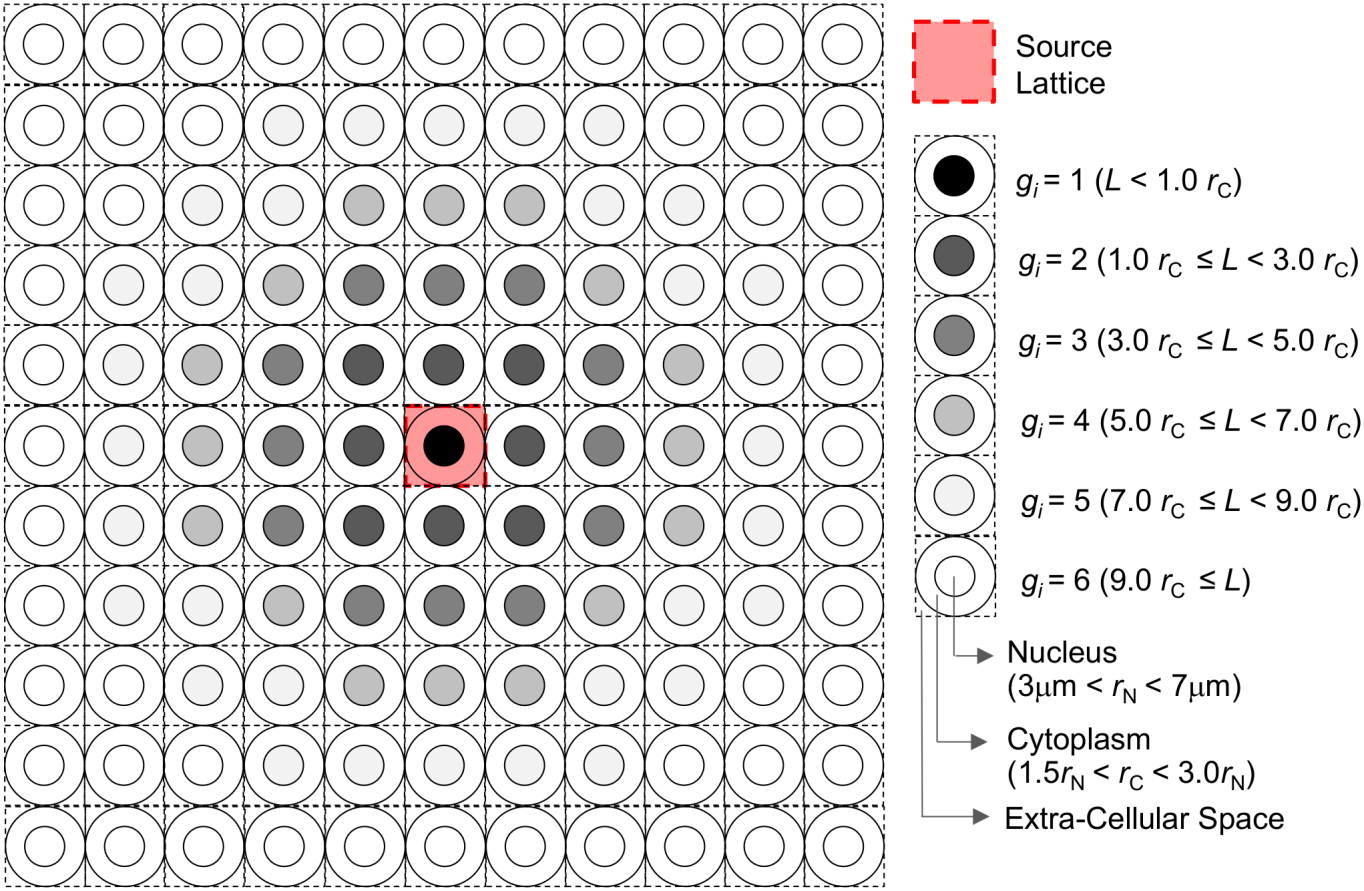
Geometry of the cell system assumed in the PHITS simulation.

The geometry of the simulations is shown in Fig. 1. Cells and their nuclei were assumed to be concentric spheres comprised of 1 g/cm^3^ of liquid water. They were placed in an 11×11×11 lattice structure, yielding 1,331 cells in the system. Cell nuclei were categorized into six groups according to the distance from their center to the origin of the cell system, *L* (Fig. 1). The cell system was infinitely surrounded by liquid water. To analyze the cell size dependence, we prepared 15 different cell systems by uniformly changing the radii of cells and their nuclei, denoted *r*_C_ and *r*_N_, respectively. The *r*_N_ was changed from 3 to 7 µm in 1 µm steps, while *r*_C_ was set to 1.5, 2, or 3 times larger than *r*_N_. For each cell system and RI source, the simulations were carried out four times by changing the RI localizations, where RIs were uniformly distributed in the cell nucleus, cytoplasm, extracellular space, or entire region of the central lattice.

In the PHITS simulations, all types of radiation were transported down to 1 keV, below which particles stop and deposit their entire energy at their location, except for positrons that cause pair production. This local approximation is adequate for our simulation because the ranges of 1 keV electrons and α particles are negligibly short compared to cell size. The probability density (PD) of the specific energy *z* in a cell nucleus, *f_i_*(*z*), was estimated by calculating the energy deposited in each cell nucleus *i* per source emission. In addition, the dose PDs in each cell nucleus *i* as a function of the lineal energy *y* for a site diameter of 1 µm, *d_i_*(*y*), were also derived from the PHITS simulation. The terminology for these microdosimetric quantities are defined in the International Commission on Radiation Units and Measurements (ICRU) Report 36 [16]. For calculating *d_i_*(*y*), the unique microdosimetric function of PHITS [17] was employed, which can directly determine microdosimetric PDs using a mathematical equation developed on the basis of track-structure simulations [18].

### Data Analysis

The calculated PDs of *z* in each cell nucleus, *f_i_*(*z*), were used to estimate the RBE due to the RI-inhomogeneity and bystander effects. On the other hand, the calculated dose PDs as a function of *y*, *d_i_*(*y*), were used to estimate the RBE due to the track-structure effect by combining the *Q*(*y*) relationship, which is the radiation quality factor expressed as a function of *y* for a site diameter of 1 µm defined in ICRU Report 40 [19]. For this estimate, we must convert the PDs for each cell nucleus to their mean value for all cell nuclei, including the contribution from cell nuclei located outside the lattice structure. The procedure for this conversion is given below.

The mean number of cell nuclei categorized in group *j* having *z*>0, *N*_G*j*_, can be calculated by
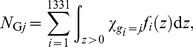
(1)where *χ* denotes the indicator function having a value of 1 only when *g_i_* = *j*, and *g_i_* is the group index for cell nucleus *i*. The single-event PDs of specific energy in a cell nucleus, *f*_1,G*j*_(*z*), were then determined by



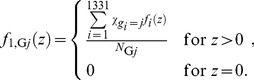
(2)Note that *f*_1_(*z*) was defined as the PD of *z* deposited in a single event in ICRU Report 36 [16], i.e., the contributions from non-hit targets are not included in *f*_1_(*z*). The frequent mean specific energy in cell nuclei categorized in group *j* having *z*>0, 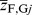, can be calculated by
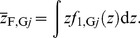
(4)


To determine the contribution from cell nuclei located outside the lattice structure, which were categorized into group 7, we assumed that the single-event PD of *z* in those outer cell nuclei is the same as that for the outmost cell-nucleus group in the lattice structure, i.e., *f*_1,G7_(*z*) = *f*_1,G6_(*z*). Consequently, 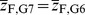. The validity of this assumption will be discussed later in this paper. Under this assumption, the mean number of the outer cell nuclei having *z*>0 per source emission, *N*_G7_, can be calculated from the ratio of the total to the mean deposition energies in the outer cell nuclei, which is written as
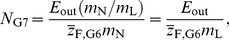
(5)where *E*_out_ is the total deposition energy outside the lattice structure, and *m*_N_ and *m*_L_ are the masses of a cell nucleus and a lattice, respectively. In this study, the value of *E*_out_ was determined by the PHITS simulation. The single-event PD of *z* in a cell nucleus averaged over the entire system, *f*_1,ave_(*z*), can be calculated by



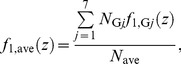
(6)where *N*_ave_ is the mean number of the cell nuclei having z>0 per source emission. Namely, 
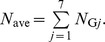


Similarly, the dose PD as a function of *y* averaged over the entire system, *d*_ave_(*y*), was also estimated from the dose PD for each cell nucleus *i*, *d_i_*(*y*), obtained from the PHITS simulation. Except for cell nuclei outside the lattice structure, the dose PD for cell nuclei categorized in group *j*, *d*_G*j*_(*y*), can be calculated by
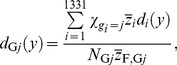
(7)


where 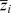 is the mean specific energy in cell nucleus i per source emission. The value of 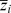 can be determined by
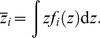
(8)


For *j* = 7, the dose PD was again assumed to be identical to the outmost cell-nucleus group in the lattice structure, i.e., *d*_G7_(*y*) = *d*_G6_(*y*). Therefore, *d*_ave_(*y*) can be calculated by
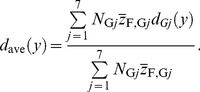
(9)


### RBE Estimation

#### RBE for the RI-inhomogeneity effect

To estimate the RBE, we assumed that radiation effects are only initiated by the ionization inside a cell nucleus; although there have been several lines of evidence that targeted cytoplasmic irradiation can induce biological effects [20], [21]. The reason for introducing this assumption is that our primary purpose is to estimate the maximum possible RBE for the internal exposure to ^137^Cs, ^134^Cs, and ^131^I, which are expected to be higher under this assumption; when RIs are localized in cell nuclei, the higher cell-nucleus dose directly results in higher RBE values.

Under this assumption, the RBE for the RI-inhomogeneity effect can be defined as the ratio of the mean specific energy in a cell nucleus to that in a lattice. When RIs are uniformly distributed in each lattice and equilibrium between the incoming and outgoing particle energies is established, the mean specific energy in a cell nucleus and in a lattice, 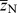 and 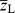, can be calculated by
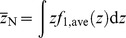
(10)


and
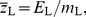
(11)respectively, where *E*_L_ is the total energy deposited inside a lattice, which corresponds to the mean source energy emitted from the RIs. Note that this RBE is equal to 1 for external exposure as well as internal exposure for the intake of RIs without any microscopic localization tendency, i.e., those uniformly distributed in all subcellular structures.

It should also be mentioned that RIs are inhomogeneously distributed inside the human body not only on the microscopic scale of subcellular structures but also on the macroscopic scale of organs and tissues. However, the influence of the RI inhomogeneity on the macroscopic scale has already been taken into account in estimating the effective dose for internal exposure by introducing biokinetic models as well as the tissue weighting factor [22]. Thus, the RBE due to macroscopic RI inhomogeneity is not discussed in this paper.

#### RBE for the track-structure effect

In this study, the influence of the track-structure was represented by the mean quality factor based on the *Q*(*y*) relationship [19], *Q*_ave_, which can be calculated by
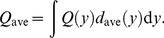
(12)


The RBE for the track-structure effect was then obtained from the ratio of *Q*_ave_ for internal exposure to the reference condition, which was represented by exposure to 662 keV photons as above.

It should be noted that the *Q*(*L*) relationship [23] is more frequently used than the *Q*(*y*) relationship in RBE calculations for radiological protection purposes [3], [24]. However, *Q*(*L*) is not designed to express the energy dependence of the RBE for electrons except for very low energy to maintain the relation as much simplicity as possible. Instead, the use of *Q*(*y*) allows RBE estimation considering the energy dependence, because the difference in RBE between γ-ray and low-energy X-ray exposures is distinguishable in the relation. Thus, we here employed *Q*(*y*).

#### RBE for the bystander effect

The bystander effect is considered attributable to the inhomogeneity of absorbed dose on microscopic scales. Thus, it should be in close connection with the PDs of *z* in cells or cell nuclei. Many studies have been devoted to develop models for quantitatively describing the bystander effect [25]–[29], but none of the existing models fully characterized the radiation fields using such microdosimetric PDs. We therefore developed an original model for describing the bystander effect based on the Fakir model [28], introducing the PDs of *z* in cell nuclei to express the dose inhomogeneity.

The followings are the hypotheses adopted in our model:

1. A cell is affected by bystander effects (e.g., those manifested as gene mutations, chromosomal aberrations, and cell killing) when receiving a bystander signal.
2. Bystander signals are emitted from irradiated cells triggered with a probability depending on irradiation conditions, but their strength is independent of these conditions;
3. The probability that a cell is not triggered after irradiated with its nucleus specific energy *z*, *S*(*z*), can be expressed by the linear-quadratic model in the same manner as its survival fraction, namely
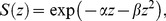
(13)where α and β are parameters that depend on radiation types and cell lines;
4. Bystander signals uniformly propagate over a certain distance;
5. All cells within the propagation distance can receive a bystander signal irrespective of whether they are directly irradiated or not; and
6. The fraction of cells receiving a bystander signal from a single signal-emitting cell is constant

Except for items 3 and 5, these hypotheses are similar to those adopted in Ref. [28], and their adequacy was discussed therein.

In the second and third hypotheses, the fraction of signal-emitting cells in the radiation field having the mean absorbed dose *D*, *P*_S_(*D*), can be calculated by
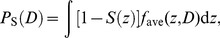
(14)where [1–*S*(*z*)] represents the triggering probability of a cell having its nucleus specific energy *z*, and *f*_ave_(*z*,*D*) denotes the averaged PD of *z* in the radiation field. Similar to the analyses given in Ref. [30], the values of *f*_ave_(*z*,*D*) can be determined by numerically solving the equation



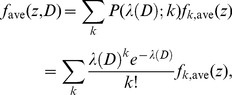
(15)where *P*(*λ*(*D*);*k*) represents the Poisson distribution with an expected value *λ*(*D*) that is the mean number of events contributing to the dose, i.e., 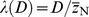. The function *f_k_*_,ave_(*z*) denotes the PD of *z* for cell nuclei irradiated by *k* events, which can be obtained from the convolution of those for cell nuclei irradiated by 1 and *k*–1 events, *f*_1,ave_(*z*) and *f_k_*_-1,ave_(*z*), respectively, expressed as
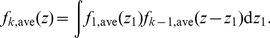
(16)


Using *f*_1,ave_(*z*) obtained from Eq. (6), the numerical values of *f_k_*_,ave_(*z*) were iteratively calculated up to when the conditions of *k*>*λ*(*D*) and *P*(*λ*(*D*);*k*)<0.0001 were satisfied, e.g., *k* = 136 for *λ*(*D*) = 100.

Let *N*_0_ be the number of cells within the distance in which the bystander signals can propagate, and *η* be the fraction of cells receiving a bystander signal from a signal-emitting cell. The probability that a cell does not receive the entire signal emitted, *P*_A_(*D*), is then given by the sum of the binomial probabilities, which is written as
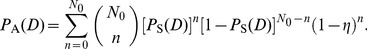
(17)


According to Ref. [28], the calculation of this sum yields 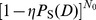. The probability that a cell receives a bystander signal, *P*_B_(*D*), which is referred to as the bystander probability in this paper, can be determined by
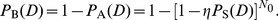
(18)


It should be noted that this bystander probability does not represent the fraction of cells that actually respond to the bystander signal. For example, the actual fraction of cells inactivated after receiving a cell-killing bystander signal is supposed to be 10∼20%, because the survival fraction of bystander cells is generally saturated around 80∼90%, even for high-dose irradiation [31], [32]. The actual fraction, however, is unlikely relevant to irradiation conditions and is hence not important in the estimation of the RBE. Thus, we defined the RBE for the bystander effect as the ratio of the absorbed dose that yields the same *P*_B_(*D*) for the internal exposures and reference condition.

## Results and Discussion

### Probability Density

**Figure 2:**
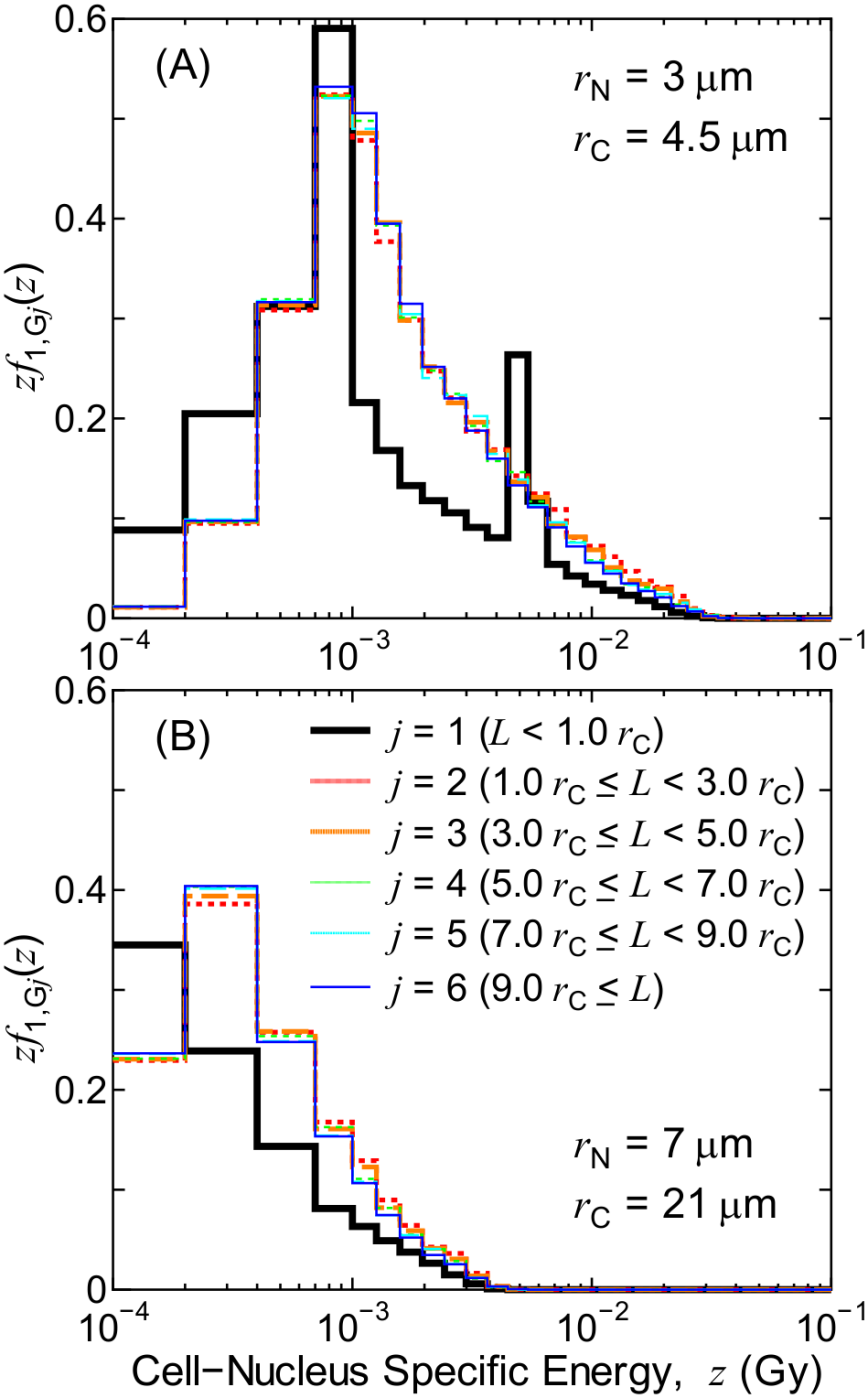
Dependence of the PDs of *z* on the cell-nucleus group *j*. These data represent the exposure to electrons emitted from ^137^Cs localized inside the cell nucleus. Panels A and B show the data for the smallest and largest cell sizes, respectively.

Figure 2 shows the calculated PDs of *z* for each cell-nucleus group *j*, *zf*_1,G*j*_(*z*), for the exposure to electrons that are emitted from ^137^Cs localized inside the cell nucleus. It should be mentioned that the PD of *z* is generally depicted in the form of *zf*(*z*) on a semi-logarithmic graph, because their integrated probabilities, *f*(*z*)d*z*, can be directly estimated from the graph by eye. These data are for the case of the smallest (*r*_N_ = 3 µm, *r*_C_ = 4.5 µm) and largest (*r*_N_ = 7 µm, *r*_C_ = 21 µm) cells. It can be found from Fig. 2 that the PDs were nearly independent of the distance from the source to the cell nucleus except for *j* = 1, the central nucleus in which the RIs were localized. This tendency can be seen in the data for other irradiation conditions, thereby verifying the assumption that *f*_1,G7_(*z*) = *f*_1,G6_(*z*). The peak observed in the PD for *j* = 1 for the smallest cell was due to the full-energy absorption of the Auger electrons around 4 keV.

**Figure 3:**
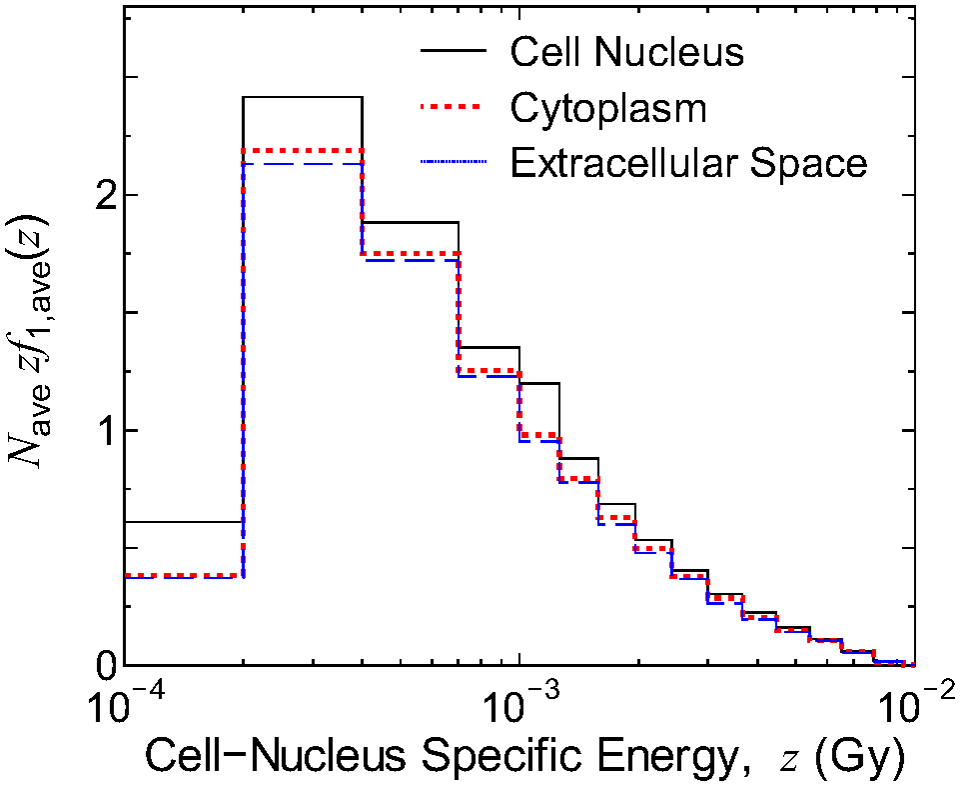
Dependence of the *N*_ave_-weighted PDs of *z* on the RI localization tendency. Data represent the exposure to electrons emitted from ^137^Cs localized inside the cell nucleus, cytoplasm, and extracellular space, respectively, for a median cell size (*r*_N_ = 5 µm, *r*_C_ = 10 µm).

Figure 3 shows the averaged PDs multiplied by the mean number of irradiated cell nuclei per source emission, *N*_ave_ *zf*_1,ave_(*z*), for exposure to electrons emitted from ^137^Cs localized in either the cell nucleus, cytoplasm, or extracellular space for the median cell size (*r*_N_ = 5 µm, *r*_C_ = 10 µm). The PDs agreed well with one another except for the very low specific energy region, where the PDs become larger when ^137^Cs was localized in the cell nucleus. This is because low-energy β-rays and Auger electrons can deposit their energy inside a cell nucleus only when they are generated inside or very close to the nucleus. The RBE for the RI inhomogeneity is attributed to this difference, as discussed later.

**Figure 4:**
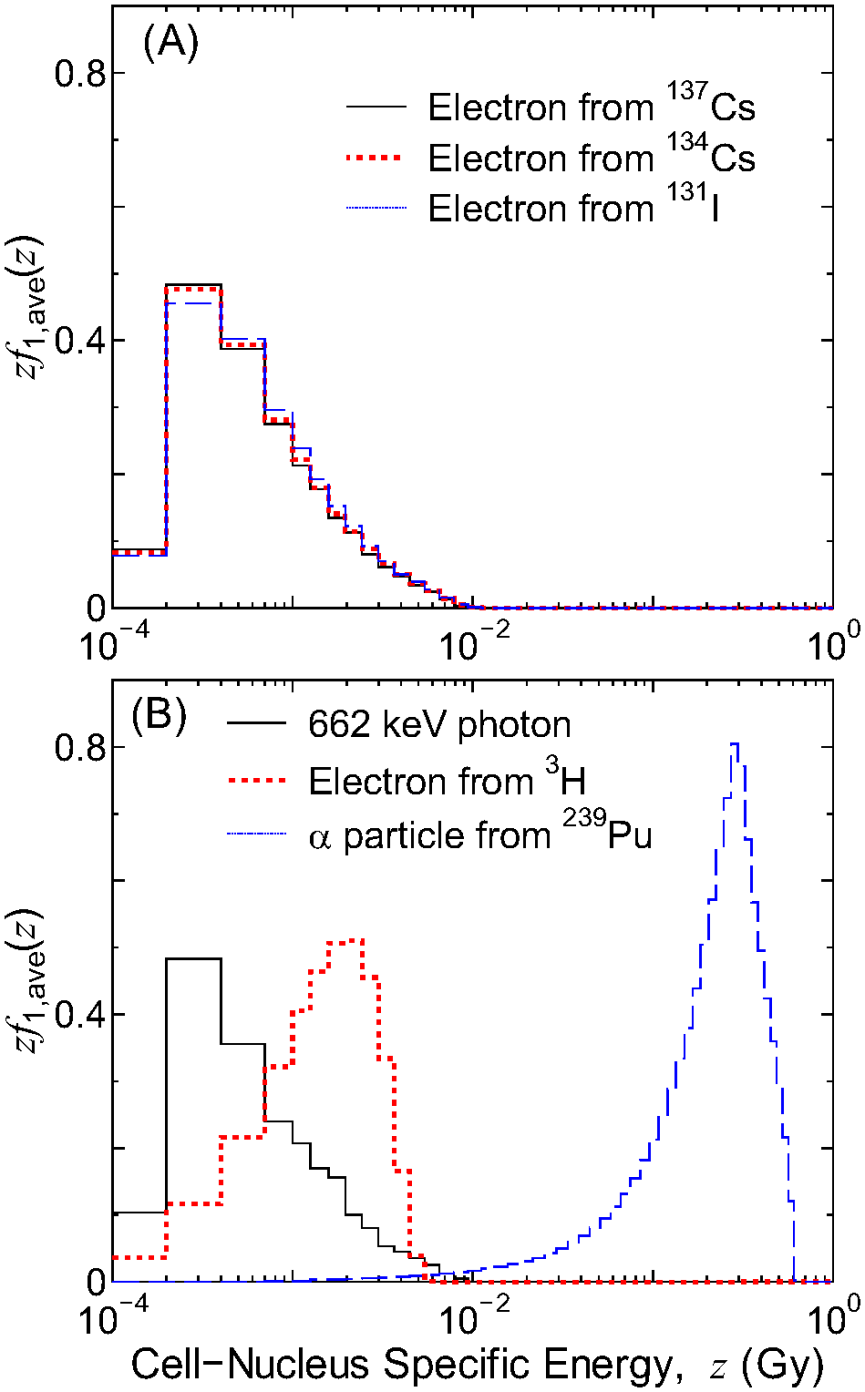
Averaged PDs of *z* for exposure to various sources uniformly distributed inside a lattice. Data are for the median cell size (*r*_N_ = 5 µm, *r*_C_ = 10 µm).

**Figure 5:**
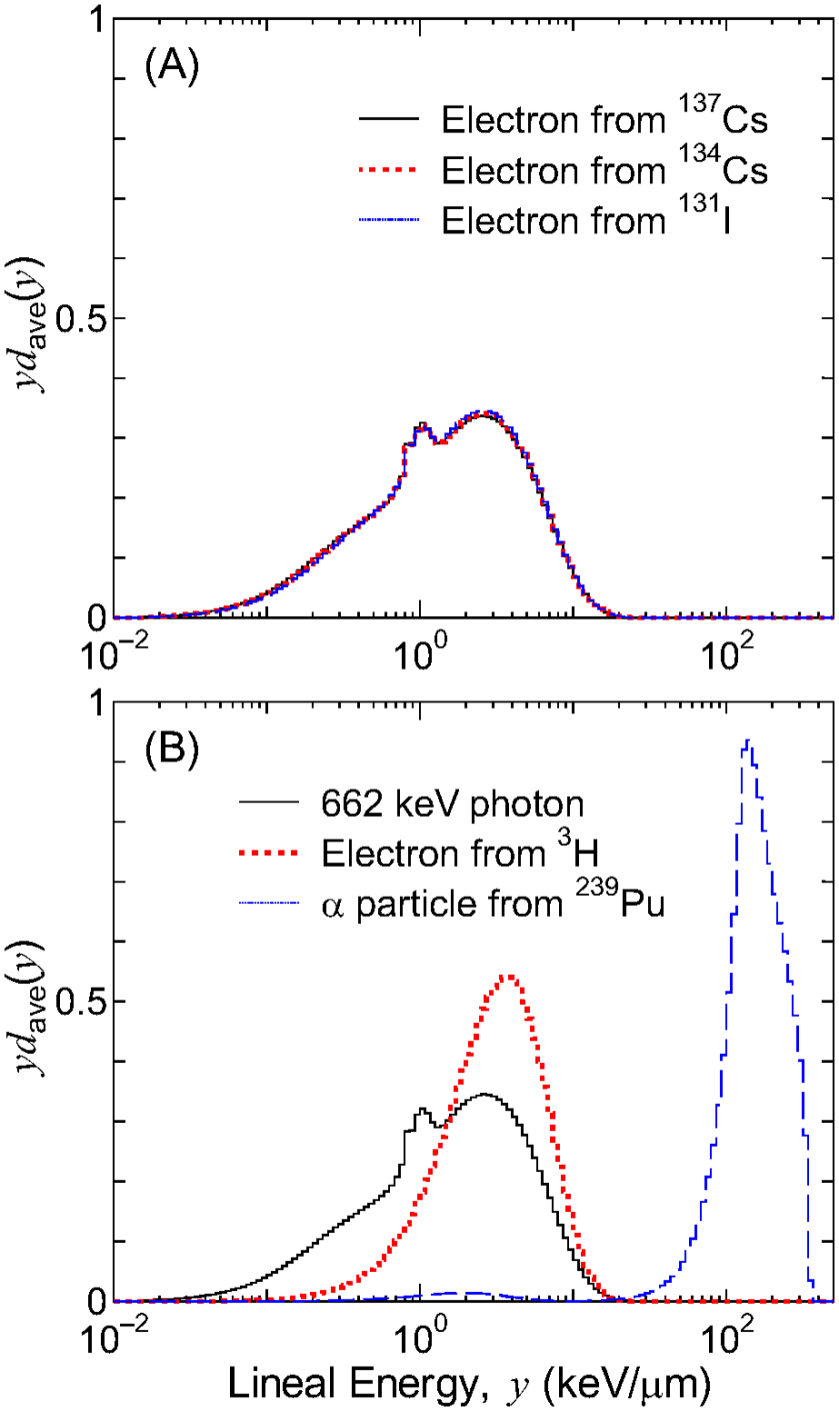
Averaged dose PDs of *y* for exposure to various sources uniformly distributed inside a lattice. Data are for the median cell size (*r*_N_ = 5 µm, *r*_C_ = 10 µm).

Figure 4 shows the averaged PDs of *z* and Figure 5 shows dose PDs of *y*, *zf*_1,ave_(*z*) and *yd*_ave_(*y*), respectively, for exposures to various sources uniformly distributed inside a lattice. The PDs for high-energy β emitters, i.e., ^137^Cs, ^134^Cs, and ^131^I, agreed well with one another, and were similar to those for 662 keV photons. Conversely, the PDs for α emitter ^239^Pu and low-energy β emitter ^3^H were shifted to higher *z* or *y* regions because of their higher stopping powers, particularly for ^239^Pu. This verifies that the absorbed dose distributions in subcellular and intranuclear scales are inhomogeneous for the intake of α and low-energy β emitters, when compared to high-energy β emitters producing the same mean absorbed dose. The difference in *zf*_1,ave_(*z*) and *yd*_ave_(*y*) results in the difference in the RBE for the bystander and track-structure effects, respectively, as discussed in the next section.

### RBE Estimation

#### RBE for the RI-inhomogeneity effect

**Figure 6:**
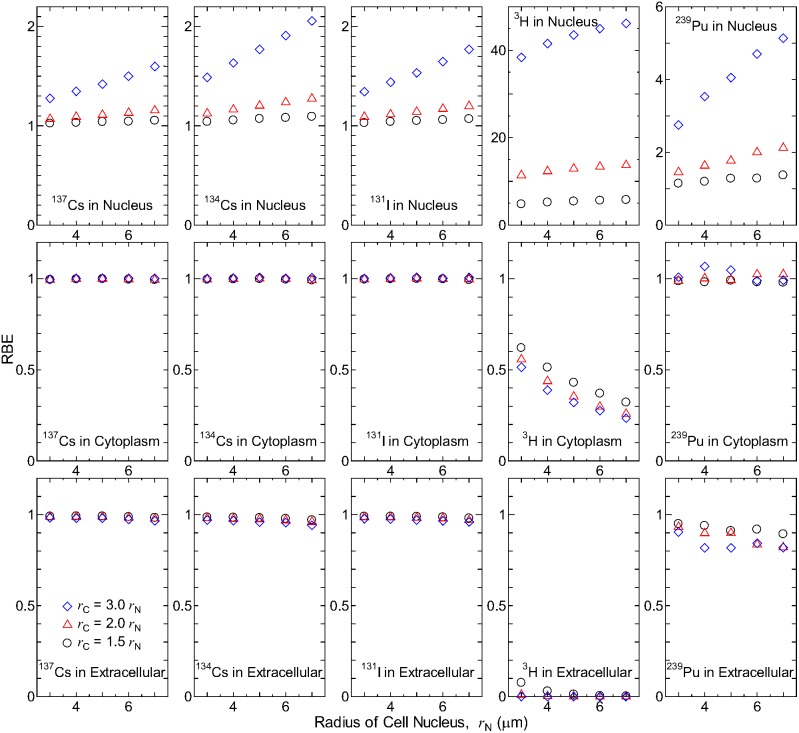
RBEs for the RI-inhomogeneity effect as a function of the cell nucleus radius. The upper, middle, and lower panels are for the cases that RIs are localized in cell nucleus, cytoplasm, and extracellular space, respectively.

Figure 6 shows the RBEs for the RI-inhomogeneity effect as a function of radius of the cell nucleus. When the RIs is localized in cell nuclei, the RBE increases with increasing radii of cells and their nuclei due to the additional deposition energy from the low-energy particles emitted inside the cell nucleus. This tendency can be expected from the PDs shown in Fig. 3. The RBE was particularly high for ^3^H and ^239^Pu due to the shorter range of the emitted particles (low-energy β and α particles, respectively). The RBE for ^134^Cs was slightly higher than that for ^137^Cs and ^131^I because of its lower mean energy of the emitted β-rays.

On the other hand, the RBE was less than or close to 1 when the RIs were localized in the cytoplasm or extracellular space because lower energy particles cannot reach the cell nucleus from such extranuclear compartments. Moreover, the RBE was nearly zero for ^3^H localized in the extracellular space, thereby indicating that the β-rays emitted from ^3^H can deposit their energy inside the nucleus only when ^3^H is incorporated into the cell. The RBE was occasionally greater than 1 for ^239^Pu localized in the cytoplasm because some of the emitted α particles create a Bragg peak inside the cell nucleus. It should be noted that the RBE was always equal to 1 when RIs were uniformly distributed inside a lattice, although this was not shown in the figure.

#### RBE for the track-structure effect

Table 1 summarizes the RBE for the track-structure effect estimated from *d*_ave_(*y*) in combination with the *Q*(*y*) relationship. These data represent the mean values and their standard deviations for all cell sizes and RI localizations analyzed in this study. Note that the standard deviations were mostly attributed to the differences in the calculation conditions because statistical uncertainties of the Monte Carlo particle transport simulations were negligibly small. Thus, the small standard deviations indicate that the RBE for the track-structure effect is not sensitive to the size of cells or the localization of RIs. As expected from Fig. 5, the estimated RBE was very close to 1 for ^137^Cs, ^134^Cs, and ^131^I, and those for ^3^H and ^239^Pu were slightly and much larger than 1, respectively.

**Table 1:** Mean RBEs and their standard deviations (SD) for the track-structure effect estimated from *d*_ave_(*y*) in combination with the *Q*(*y*) relationship.

RI	Mean RBE	SD	P-value
^137^Cs	1.00	0.014	0.40
^134^Cs	1.02	0.036	0.028
^131^I	1.02	0.016	0.035
^3^H	1.49	0.000	0.000
^239^Pu	37.0	0.26	0.000

The p-values obtained from Welch’s t-test between the average quality factors for each internal exposure and the reference condition are also given.

It should be mentioned that the average quality factors, *Q*_ave_, were smaller than the data given in Table 1 by a factor of 0.69, which is the value of *Q*_ave_ for the reference condition (662 keV photon exposure). This is because the reference radiation of the *Q*(*y*) relationship was set to low-energy X-rays whose RBE was generally considered to be higher than that of γ-rays. The p-values obtained from Welch’s t-test between *Q*_ave_ for each internal exposure and the reference condition are also given in Table 1. Except for ^137^Cs, the p-values were smaller than the significance level; p<0.05. This result indicates that their RBE values are greater than 1 in statistically significant.

#### RBE for the bystander effect

Our bystander model contains four free parameters: *α*, *β*, *N*_0_, and *η*. These parameters should have a complicated dependence on the biological endpoint and the cell type, and the evaluation of their numerical values is outside the scope of this paper. Thus, we estimated the RBE for the bystander effect for various conditions by arbitrarily changing the parameters.

**Figure 7:**
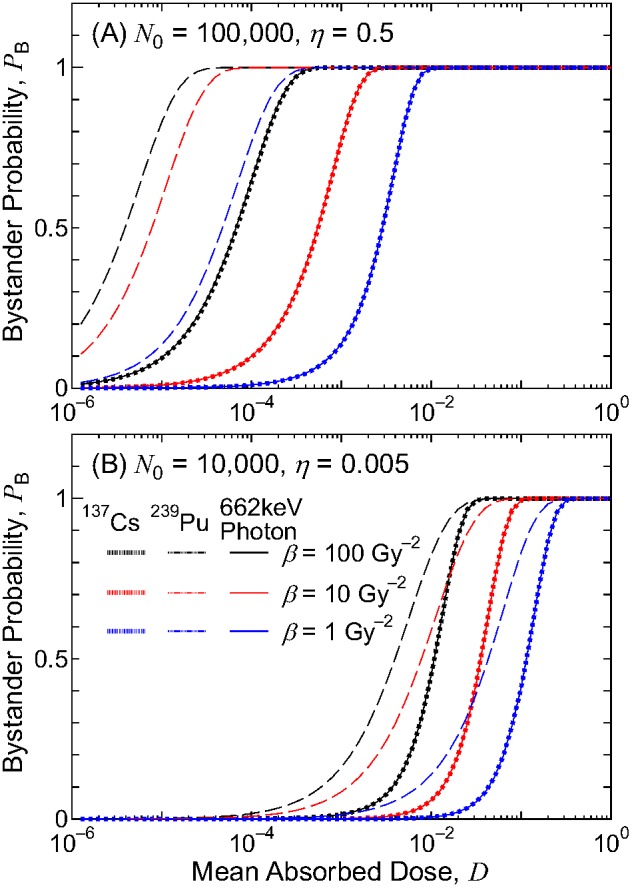
Bystander probabilities for various irradiation conditions as a function of the mean absorbed dose *D*. Panels A and B show the results for the reactive (*N*_0_ = 100,000 and *η* = 0.5) and unreactive (*N*_0_ = 10,000 and *η* = 0.005) bystander signals, respectively.

As examples, the bystander probabilities for exposure to electrons from ^137^Cs, α particles from ^239^Pu, and 662 keV photons are shown in Fig. 7 for two different parameter settings: *N*_0_ = 100,000 and *η* = 0.5 as well as *N*_0_ = 10,000 and *η* = 0.005. These conditions represent the reactive and unreactive bystander signals, where approximately 0.001% and 1% of cells, respectively, must be initiated in order to fully induce the bystander effect. For both conditions, the *α* parameter was set to 0 to express the threshold behavior of the triggering probability, whereas the *β* parameter was changed from 1 to 100 Gy^−2^ to investigate the dependence of the RBE on the threshold specific energy. These data were similar irrespective of whether the RIs were uniformly distributed inside a lattice or had a microscopic localization tendency.

Nearly perfect agreements between the bystander probabilities for the exposures to electrons from ^137^Cs and 662 keV photons were observed in Fig. 7 for all parameter settings. The corresponding data for the exposure to electrons from ^134^Cs and ^131^I also agreed with the photon data, although not shown in the graph. These agreements were attributed to the fact that their radiation fields characterized by *f*_1,ave_(*z*) were nearly identical to one another, as shown in Fig. 4. Thus, the RBE for the bystander effect for exposure to ^137^Cs, ^134^Cs, and ^131^I should be very close to 1 irrespective of the calculation conditions.

**Figure 8:**
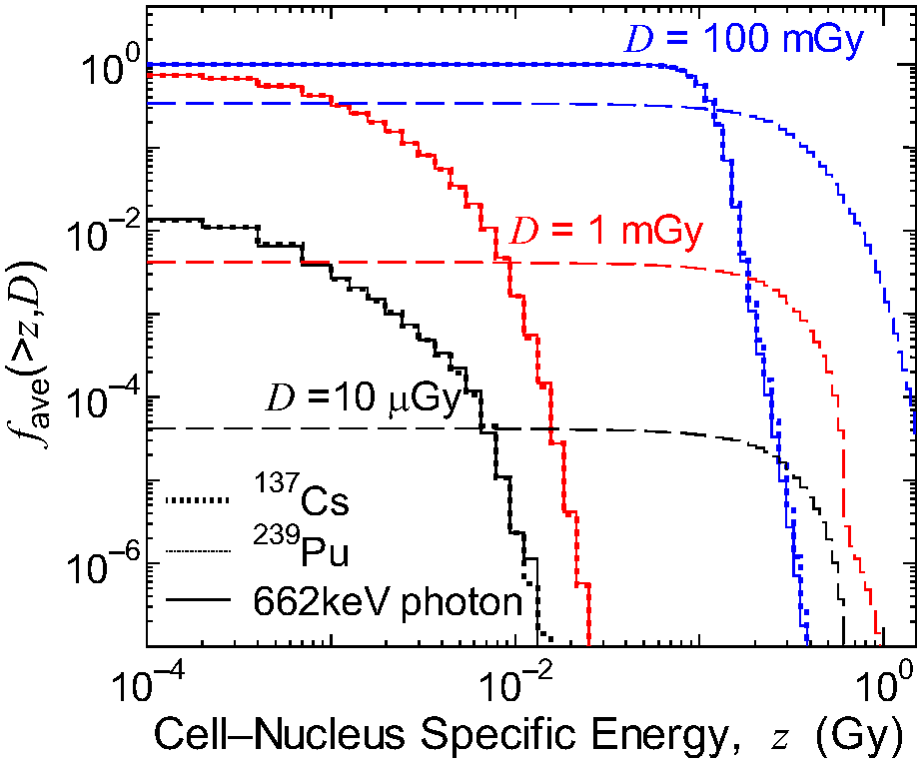
Probabilities of cell nuclei having the specific energy over *z* in various radiation fields, *f*_ave_(>*z*,*D*). Data are for exposure to electrons from ^137^Cs, α particles from ^239^Pu, and 662 keV photons with mean absorbed dose *D* = 10 µGy, 1 mGy and 100 mGy, respectively.

Conversely, the bystander probability for the exposure to α particles from ^239^Pu was significantly higher than that for the electron and photon exposures at the same absorbed dose. This tendency can be explained by the probability of cell nuclei having the specific energy over *z* in those radiation fields with the mean absorbed dose *D*, *f*_ave_(>*z*,*D*), where their examples are shown in Fig. 8. For electron or photon exposures with *D* = 10 µGy, approximately 1% of the cell nuclei were irradiated and their maximum specific energy was around 10 mGy. Thus, their bystander probabilities were very small, even for the largest *β* parameter, i.e., the lowest threshold specific energy. On the other hand, only 0.004% of the cell nuclei were irradiated for a 10 µGy exposure to α particles, but most irradiated cell nuclei had a specific energy >100 mGy. Thus, the bystander effect is induced even by such low-dose exposure for the case of the reactive bystander signal. Consequently, the RBE for the bystander effect for exposure to ^239^Pu is generally >1, and depends on both the model parameters and mean absorbed dose in a complicated manner.

#### Maximum possible RBE

**Figure 9:**
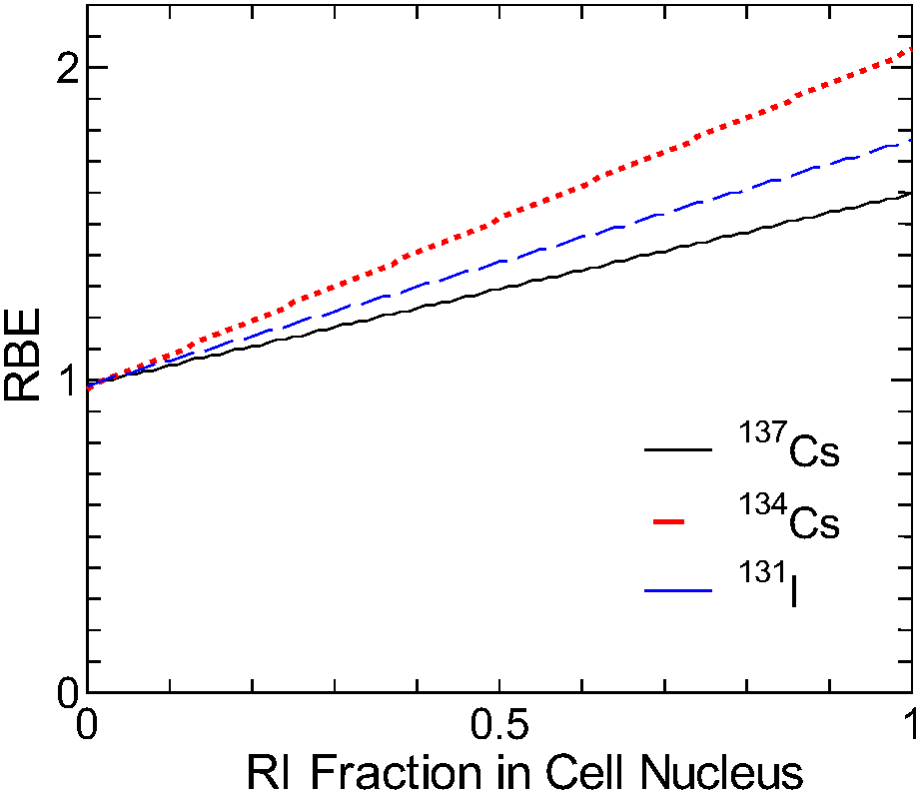
RBEs for the RI-inhomogeneity effect as a function of the RI fraction inside cell nuclei. Data are for exposure to electrons emitted from ^137^Cs, ^134^Cs, and ^131^I, and for the largest cell size (*r*_N_ = 7 µm, *r*_C_ = 21 µm).

It is evident from the above analyses that the RI-inhomogeneity effect is the dominant factor in determining the RBE for internal exposure to ^137^Cs, ^134^Cs, and ^131^I. In this subsection, their maximum possible RBE was estimated considering the realistic RI localization tendency. Figure 9 shows the calculated RBE for the RI-inhomogeneity effect for exposure to electrons emitted from ^137^Cs, ^134^Cs, and ^131^I as a function of the RI fraction in cell nuclei. In this calculation, the rest of RIs was assumed to be uniformly distributed in the cytoplasm and extracellular space. These data were for the largest cell size (*r*_N_ = 7 µm, *r*_C_ = 21 µm), which yielded the highest RBE as shown in Fig. 6. The RBE linearly increased with increasing RI fraction.

According to an animal study in Ref. [9], 12–21% of ^137^Cs were localized inside the cell nuclei. If the maximum fraction of ^137^Cs and ^134^Cs in nuclei of human cells is also 21%, then the maximum possible RBE for exposure to electrons from those RI will be ∼1.1 and ∼1.2, respectively. This estimate is quite conservative because cell-nucleus mass adopted in this calculation was very small – only 2% of the total weight. Thus, intranuclear cesium concentration is approximately 10 times higher than that in the other structures. This value is quite high compared to the results obtained with the yeast *Saccharomyces* [33], where intranuclear cesium concentration and cytoplasmic one were nearly equal. In addition, the electron contributions to the effective dose were estimated to be only 43% and 16% due to the intake of ^137^Cs and ^134^Cs, respectively, on the basis of the specific absorbed fractions calculated using the ICRP/ICRU adult male reference phantom [34] in combination with a physiologically based biokinetic model for cesium in human body [35]. The rest is the contribution from photons whose RBE is equal to 1, and thus, the maximum possible RBE for the intake of ^137^Cs and ^134^Cs including the photon contribution were approximately 1.04 and 1.03, respectively.

For the exposure to ^131^I, there is no evidence that thyroid cell nucleus accumulates iodine, although its subcellular distribution has not been thoroughly revealed. In addition, the extracellular space such as lumen and blood contains a non-trivial portion of iodine [36]. On the other hand, the thyroid hormones containing iodine tend to bind to nuclear receptors in cells of extrathyroidal tissues such as liver and kidney. However, the organ absorbed dose in these tissues due to the intake of ^131^I was much smaller than that in thyroid, which accounted for ∼98% of the effective dose. Thus, the assumption that iodine is uniformly distributed inside the cell nuclei probably yields an adequate estimate of the RBE due to the intake of ^131^I. Even if the intranuclear iodine concentration is twice as high as that of the entire system, the RBE would only be 1.02.

### Conclusions

The PDs of specific energy inside cell nuclei and dose PDs of the lineal energy for a site diameter of 1 µm for internal exposure to ^137^Cs, ^134^Cs, and ^131^I, as well as external exposure to 662 keV photons, were calculated by performing Monte Carlo particle transport simulations with PHITS. The RBEs for the RI-inhomogeneity, track-structure, and bystander effects were then derived from the calculated PDs. The RBEs for the track-structure and bystander effects were very close to 1, owing to the nearly identical PDs between the internal exposure and the external exposure to 662 keV photons. On the other hand, the RBEs for the RI-inhomogeneity effect largely depended on the intranuclear RI concentration and cell size. However, their maximum possible RBE was only 1.04 even under conservative assumptions. Therefore, it can be concluded from the microdosimetric viewpoint that the risk from internal exposure to these RIs should be nearly equivalent to that from external exposure to γ-rays at the same absorbed dose level, as suggested in the current ICRP recommendations [22].
